# Planktonic and epilithic prokaryota community compositions in a large temperate river reflect climate change related seasonal shifts

**DOI:** 10.1371/journal.pone.0292057

**Published:** 2023-09-21

**Authors:** Attila I. Engloner, Márta Vargha, Péter Kós, Andrea K. Borsodi

**Affiliations:** 1 Centre for Ecological Research, Eötvös Loránd Research Network, Budapest, Hungary; 2 Department of Public Health Laboratories, National Public Health Centre, Budapest, Hungary; 3 Institute of Plant Biology, Biological Research Centre, Eötvös Loránd Research Network, Szeged, Hungary; 4 Department of Biotechnology, Faculty of Science and Informatics, Szeged University, Szeged, Hungary; 5 Department of Microbiology, ELTE Eötvös Loránd University, Budapest, Hungary; Université de Perpignan: Universite de Perpignan Via Domitia, FRANCE

## Abstract

In freshwaters, microbial communities are of outstanding importance both from ecological and public health perspectives, however, they are threatened by the impact of global warming. To reveal how different prokaryotic communities in a large temperate river respond to environment conditions related to climate change, the present study provides the first detailed insight into the composition and spatial and year-round temporal variations of planktonic and epilithic prokaryotic community. Microbial diversity was studied using high-throughput next generation amplicon sequencing. Sampling was carried out monthly in the midstream and the littoral zone of the Danube, upstream and downstream from a large urban area. Result demonstrated that river habitats predominantly determine the taxonomic composition of the microbiota; diverse and well-differentiated microbial communities developed in water and epilithon, with higher variance in the latter. The composition of bacterioplankton clearly followed the prolongation of the summer resulting from climate change, while the epilithon community was less responsive. Rising water temperatures was associated with increased abundances of many taxa (such as phylum *Actinobacteria*, class *Gammaproteobacteria* and orders *Synechococcales*, *Alteromonadales*, *Chitinophagales*, *Pseudomonadales*, *Rhizobiales* and *Xanthomonadales*), and the composition of the microbiota also reflected changes of several further environmental factors (such as turbidity, TOC, electric conductivity, pH and the concentration of phosphate, sulphate, nitrate, total nitrogen and the dissolved oxygen). The results indicate that shift in microbial community responding to changing environment may be of crucial importance in the decomposition of organic compounds (including pollutants and xenobiotics), the transformation and accumulation of heavy metals and the occurrence of pathogens or antimicrobial resistant organisms.

## Introduction

Climate change in temperate regions is reflected both in long-term environmental warming and in shifting seasons, such as prolonged summers [1]. In rivers, this trend manifests in rising water temperatures either year-round or seasonally, for example in autumn [2–4]. The impacts of global warming have been addressed by numerous research, based on both physical, chemical and ecological processes. For the latter, special attention must be paid to how environmental changes affect living organisms, especially those that play a key role in an ecosystem. In freshwaters, microbial communities are of major importance both from ecological and public health perspectives. They are essential in aquatic food webs and perform key processes in nutrient cycles e.g. by decomposing organic compounds or transforming nitrogen and sulphur compounds [5, 6]. Freshwater microbial communities can also have significant, negative or positive impact on human health. As a potential source of pathogens or antimicrobial resistant organisms, they can cause public health concerns in recreational waters and can pose a threat to drinking water quality [7], but can also contribute to the removal of organic micropollutants or heavy metals [8–10]. Taking these considerations into account, it is clear that changes in microbial communities responding to changing environment can be important in many areas. In water bodies used in multiple ways, such as large rivers which provide diverse ecosystem services and water for various purposes of the human society, the impact of these changes could be many times greater.

Microbial diversity in aquatic ecosystems depends on various physical and chemical factors, such as water temperature, organic matter, total nitrogen and phosphorus, dissolved oxygen and pH [11–13]. Therefore, assessment of bacterial diversity also helps to understand the effects of environmental pollution on river ecosystems [13–15].

Due to the increasing use of next generation sequencing (NGS) techniques, we have a broadening knowledge of the spatial diversity of microbial communities in large rivers; not only for single locations but also for multiple river sections or even along the whole length or networks of rivers [16–19]. Our knowledge on the temporal dynamics of microbial communities in rivers, however, is limited, but see Akinwole et al. and Hullar et al. [20, 21]. Studies primarily focus on one season [22] or compare the differences in microbiota between spring-autumn, summer-winter or dry-wet seasons [19, 23–25]. In a changing climate, however, seasons can become irregular, i.e. their onset, length and temperature may alter [1]. Therefore, sampling once in a three-month period may not adequately reflect a season and, in particular, the variability during the year. Although exploration of the latter requires systematic, year-round, high-frequency sampling, these studies are scarce [26, 27].

Further deficiency in our knowledge arises from the fact that river microbiota is mostly studied in water. Less data is available on microbial communities in sediment [16, 25, 28] and very few on those forming biofilm on gravel (epilithon) in the river bed [29]. Due to its filtering capacity, microbial biofilm formed in the gravel beds of rivers plays a particularly important role in the self-purification processes of waters and in providing drinking water in urbanized regions [30, 31].

In this study, changes of water quality and spatial and temporal diversity of planktonic and epilithic prokaryotes of Danube were analysed to explore how changing environment, including changes in temperature patterns related to climate change may affect these organisms. The year-round investigations primarily aimed to reveal (i) the differences in the community structure in the plankton and gravel biofilm (epilithon), (ii) major habitat conditions that may affect the occurrence of bacteria during the year and (iii) seasonal variability of the microbial communities reflecting the climate change. Furthermore, potential impact of a large metropolitan area (Budapest) and distance from the riverbank on microbial composition were also studied.

## Materials and methods

### Sampling and in situ measurements

Two study areas were designated on river Danube, on the catchment of the bank filtration drinking water abstraction sites of Budapest, the capital of Hungary. One area was located upstream (between 1678 and 1674 river km, 47°45’39.68"N 19° 7’50.24"E and 47°43’42.60"N 19° 7’42.66"E) and the other downstream (between 1607 and 1604 river km, 47°11’23.73"N 18°52’48.69"E and 47°10’5.58"N 18°52’32.34"E) from the capital. Sampling was performed every month from February 2019 to January 2020, along three transects perpendicular to the shore per study areas. River water level information was obtained from the General Directorate of Water Management.

In situ physical-chemical measurements were performed midstream and near the bank (i.e. in the littoral zone) in each transect, at a water depth of approximately 50 cm. Water temperature, pH and electrical conductivity were recorded by a Combo pH/EC/TDS/Temperature tester (HI 98129). Dissolved oxygen, redox potential and turbidity were measured by a portable dissolved oxygen meter (HI 9142), pH/Ion meter (WTW ProfiLine pH/ION 3310) and turbidity meter (Lovibond TB210), respectively.

For microbiological studies and laboratory chemical analysis, water samples were taken at the same sampling points, from a water depth of approximately 50 cm. Samples for DNA extraction were collected into sterile 1 L glass flasks by immersion.

Sampling for epilithon analysis was performed from March 2019 to January 2020. Pebbles were collected by benthological dredging, from three points per transects at water depths of 1, 2 and 5 meters, and these subsamples were combined into a single composite sample per transect. Approximately 20 pebbles 2–5 cm in diameter were collected from each transect and placed into single-use plastic bags. All samples were transported and stored at 6–8°C in cooled containers until laboratory processing within 24 hours.

### Laboratory chemical analyses and sample preparation for microbiological examinations

To further characterize the aquatic environment, NO_3_^-^ and SO_4_^2-^ concentrations of water samples were determined by ion chromatography (Dionex ICS 5000, Thermo Scientific, USA), and ortho-phosphate (PO_4_^3-^) and total phosphorus (TP) concentrations were determined by Spekord 210 Plus spectrophotometer (Analytik Jena, Germany), following [32]. Total organic carbon (TOC), as well as total nitrogen (TN) concentrations, were determined by applying a Multi N/C 2100S TC-TN analyzer (Analytik Jena, Germany) equipped with a non-dispersive infrared detector and a chemiluminescent detector, in accordance with the corresponding international standards (MSZ EN 1484:1998, MSZ EN 12260:2004).

For molecular microbiological investigations, 1L water samples were concentrated by filtration on 0.22-μm pore sized polycarbonate filters (Millipore, Billerica, MA, USA). The filters were stored at −20°C until DNA extraction. Epilithon samples were washed from pebbles into saline solutions using sterile paintbrushes. After sedimentation of suspension by centrifugation (4 000rpm, 5 min), 50 mg biofilm matter was used for DNA extraction.

### DNA extraction and Illumina sequencing

Community DNA was extracted from the concentrated water and epilithon samples using DNeasy Power Soil Kit (QIAGEN, Hilde, Germany) according to the manufacturer’s instructions. The concentration of the DNA samples was measured using Qubit 4 fluorometer (Thermo Fisher Scientific, USA). The V3-V4 region of the 16S rRNA gene was amplified using Pro34F and Pro805R Illumina primers designed for simultaneous detection of both bacterial and archaeal sequences [33] using 20 ng DNA template. The amplicon libraries were inspected and quantitated using Agilent 2100 Bioanalyzer System (Agilent Technologies, Inc., USA). The amplicons were sequenced on Illumina MiSeq platform (Illumina, San Diego, California, USA) using MiSeq Reagent Kit v3 providing 300 base long reads. The raw reads were analyzed using Qiime2 software suite [34]. The sequence pairs were joined using the vsearch plugin providing 500 bp amplicon sequences. Quality filtering was done using q-score module for three consecutive bases with Phred score less than 20. Dereplication of the so generated sequences also the vsearch plugin was used [35]. From 10088015 read pairs 7959568 high quality joined sequences were retained after quality filtering, chimera search and clipping, with an average of 19180 sequences per sample.

Subsequently, de novo OTU (Operational taxonomic unit) picking was carried out using the vsearch modul using 97% identity threshold. The OTUs were filtered to 0.005% read coverages according to the recommendations of Bokulich et al. [36]. From this dataset, the OTUs were combined to different taxonomy levels when needed and further filtering was applied.

In order to see if the sampling depth was satisfactory for the samples to represent the compositions of the entire populations, the observed OTUs were calculated and visualized using the alfa rarefaction pipeline in Qiime2. Beta diversity data were calculated using the diversity modul’s appropriate plugins.

Taxonomy was assigned using sklearn method (https://www.jmlr.org/papers/v12/pedregosa11a.html) and the ARB-SILVA SSU v.138 database (https://www.arb-silva.de/). The 1502 OTUs were classified into 711 different species, 369 genera, 197 families, 115 orders, and 43 classes.

The sequences in fastq format are deposited in NCBI as BioProject: PRJNA838445. The data will become publicly available upon the acceptance of the manuscript.

### Statistical analyses

The OTU abundances in the samples were established as read counts and normalized to relative abundances as per cent of the number of reads in each sample. For alpha diversity analysis the observed OTUs within each habitat were calculated and visualized using the alpha rarefaction pipeline in Qiime2 by summing up the observed features (OTUs) of the rarefied samples belonging to the given habitats. Beta diversity of the samples was compared as a principal coordinates analysis (PCoA) calculated from the weighted unifrac distances of the relative abundances using the beta diversity module and it was visualized using the Emperor plugin of Qiime2.

Environmental variables and relative abundance data of all order-level OTUs were evaluated by canonical variates analysis (CVA) and standardized principal component analysis (PCA) using SYN-TAX 2000 computer program package [37]. Abundant bacterial taxa were also analyzed separately; following [38], the percentage distribution of relative abundance of bacterial phyla with a mean relative abundance ≥1% were calculated, and the relative abundances of orders with a mean relative abundance ≥0.1% were evaluated by hierarchical clustering (unweighted pair group method, UPGMA, based on Bray–Curtis similarity index) and redundancy analysis (RDA). Kruskal-Wallis and post hoc Dunn’s tests with Bonferroni correction were carried out to test significant differences. Differences with p values under 0.05 were considered significant.

## Results

### Characterization of physical and chemical variables of the water

**Fig 1:**
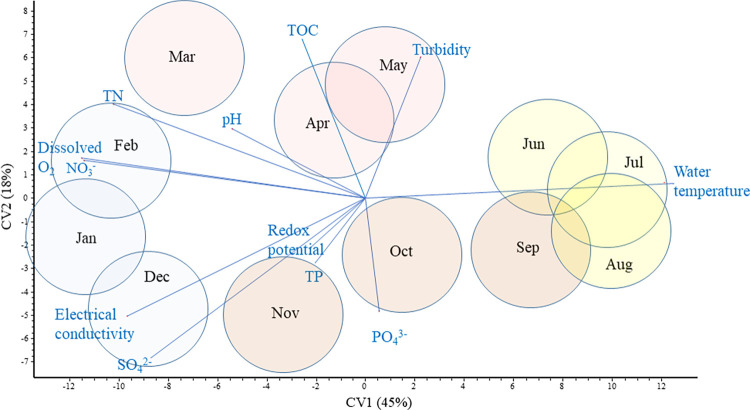
CVA biplots with 95% isodensity circles based on monthly environmental data.

Water samples clearly separated based on their physical and chemical characteristics, showing a clear seasonal pattern (Fig 1 and Table 1). However, this separation did not align completely to the four equal-length calendar seasons. From June to September water temperature was the most important environmental driver, this being the warmest period of the year. Turbidity and TOC were the highest, while concentration of phosphate was the lowest in April and May. Electric conductivity and the concentrations of sulphate, nitrate, dissolved oxygen and the total nitrogen peaked during the period from November to March.

**Table 1:** Environmental parameters of Danube water during the study year (average ± SD, n = 12 per sampling time).

	Water depth min-max (mBf)	Water temperature (°C)	Electrical conductivity (mS)	pH	Turbidity (ntu)	Dissolved O_2_ (mg/L)	Redox potential (mV)	TOC (mg/L)	TN (mg/L)	TP (μg/L)	NO_3_^-^ (mg/L)	PO_4_^3-^ (μg/L)	SO_4_^2-^ (mg/L)
**Feb**	98.75–100.76	4.13±0.54	0.43±0.01	7.92±0.21	6.17±1.61	11.81±0.39	135.83±17.86	1.89±0.35	3.02±0.12	199.90±90.87	11.05±0.08	107.67±25.51	36.37±0.56
**March**	98.78–100.34	6.29±0.19	0.34±0.03	8.03±0.22	52.60±38.51	11.18±0.28	130.47±97.40	2.82±0.85	2.70±0.21	134.75±42.13	9.96±0.74	124.17±38.89	26.31±2.74
**Apr**	99.7–102	11.32±1.41	0.32±0.01	8.00±0.14	18.53±9.03	10.04±0.41	36.01±22.21	2.07±0.69	1.91±0.32	90.86±43.96	6.98±1.34	70.28±46.07	25.88±1.05
**May**	99.22–100.21	13.31±0.89	0.29±0.01	7.67±0.23	58.98±47.88	9.88±0.66	77.63±64.81	3.01±1.30	1.81±0.37	66.23±37.00	6.24±0.86	108.33±64.65	20.93±2.83
**June**	99.61–102.86	19.75±0.69	0.26±0.00	7.54±0.21	60.54±18.94	8.14±0.38	94.95±22.07	1.87±0.19	1.26±0.13	88.73±17.14	4.82±0.44	137.50±22.70	20.25±0.61
**July**	100.09–103.04	21.69±0.56	0.27±0.02	7.44±0.16	25.64±23.98	8.45±0.45	57.70±4.68	1.47±0.23	1.12±0.24	110.28±46.14	3.72±0.13	120.83±36.23	23.19±2.09
**Aug**	98.52–100.09	21.99±0.53	0.29±0.02	7.59±0.11	23.05±19.10	8.24±0.28	76.97±22.71	1.38±0.32	1.20±0.14	117.20±38.39	3.84±0.32	152.50±48.30	25.14±0.79
**Sept**	98.59–99.94	19.23±2.50	0.32±0.01	7.51±0.30	15.81±9.06	8.59±0.39	119.30±20.98	1.62±0.17	1.35±0.09	144.82±40.83	5.00±0.29	144.00±25.13	27.38±1.96
**Oct**	97.99–99.73	13.42±1.41	0.35±0.01	7.36±0.26	13.12±8.20	9.50±0.37	123.27±30.95	1.66±0.25	1.54±0.19	119.07±41.26	5.70±0.46	119.00±15.83	28.45±1.48
**Nov**	97.87–99.27	8.22±1.69	0.39±0.04	7.94±0.47	14.33±13.38	9.80±0.26	106.66±9.52	1.72±0.31	1.77±0.15	142.00±25.32	6.55±0.42	149.58±21.77	30.99±1.42
**Dec**	97.99–99.72	3.71±0.32	0.38±0.01	7.64±0.54	10.09±3.75	10.76±0.16	93.99±3.70	1.60±0.11	1.90±0.12	111.92±25.67	9.09±0.30	154.17±31.20	34.69±1.24
**Jan**	98.13–99.91	1.64±0.29	0.39±0.01	7.93±0.19	7.39±2.61	11.83±0.34	101.32±3.40	1.70±0.27	2.35±0.07	125.54±28.46	10.17±0.08	138.75±23.09	35.78±1.72

The pH and concentrations of total phosphorus, ortho-phosphate and sulphate were higher downstream than upstream of the capital city on most sampling occasions, though the differences were only significant in a few cases (S1 Table). Monthly chemical measurements recorded in midstream and near the bank did not differ significantly either.

### Characterization of prokaryotic communities

After filtering, the retained 7 959 568 reads were assigned to 711 and 115 OTUs at seventh and fourth taxonomic (i.e. species and order) levels, respectively. The 115 order-level OTUs, the number of reads and also some relevant genera and species assigned to the OTUs are listed in S2 Table. Rarefaction curves using observed OTU numbers (S1 Fig) show that the sequencing well represented the composition of the microbial consortia. Diversity of the epilithon samples was consistently higher than that of the planktonic samples.

**Fig 2:**
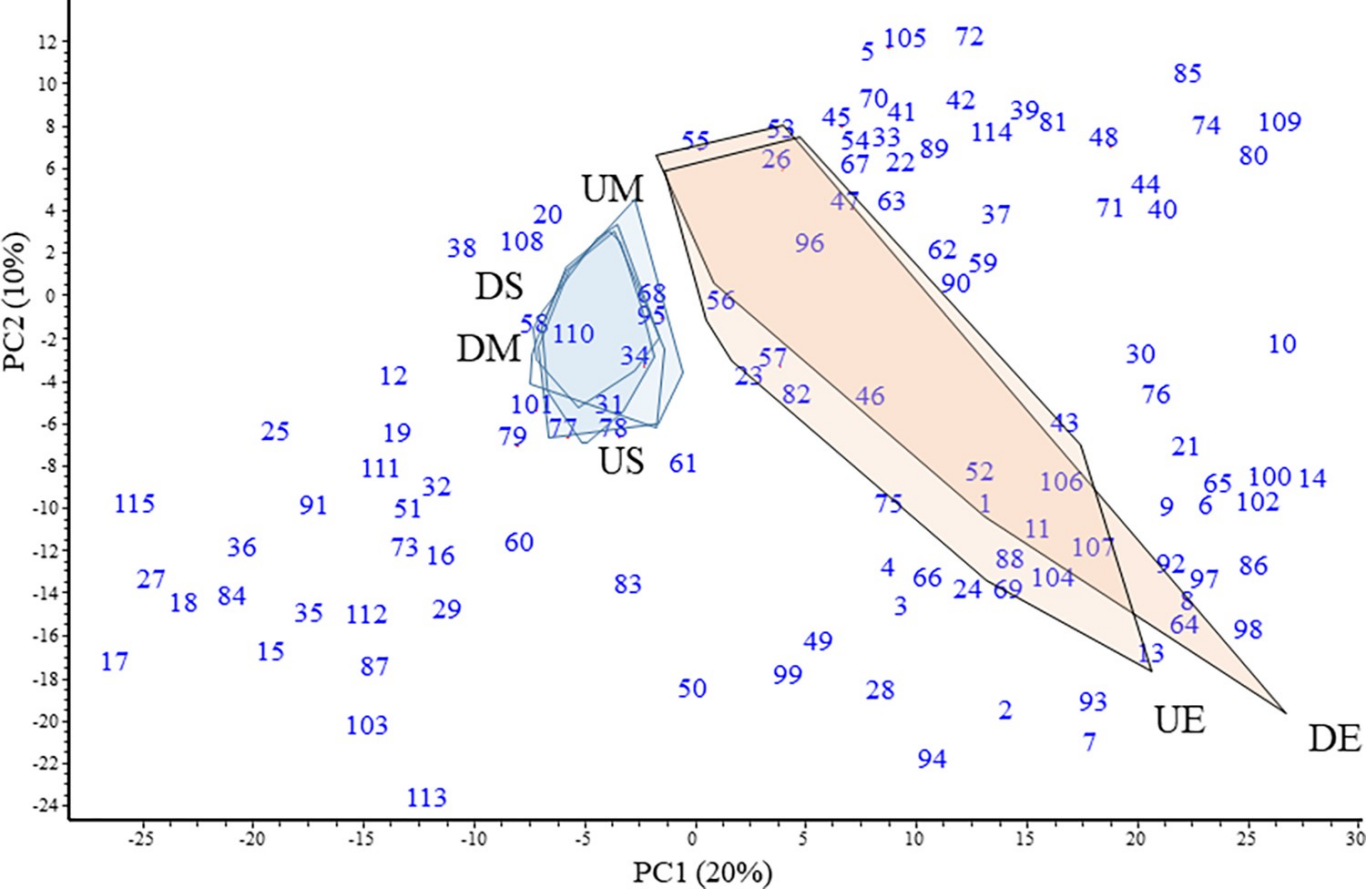
PCA ordination of water and epilithon samples based on relative abundance of amplicon sequences at order-level. Convex polygons represent samples taken upstream (U) or downstream (D), from midstream water (M), shore water (S) or from the epilithon (E). Order-level OTUs are shown as numbers; taxonomic names are listed in S2 Table.

Water and epilithon samples were well distinguished based on prokaryotic community composition with higher variance in the latter (Fig 2). Samples taken upstream and downstream from Budapest or the ones collected in the middle of the river and near the shore were highly similar, without any clear spatial discrimination. (See also S2 Fig).

Comparing the two main habitat types, more orders were positively correlated with epilithon than with water samples (see also S2 Table). Besides Bacteria, four archaeal orders were also identified: *Bathyarchaeia* and *Nitrosopumilales* of the phylum *Crenarcheota* and *Methanomicrobiales* and *Methanosarcinales* of the phylum *Halobacterota*. All detected archaeal taxa were associated with the epilithon samples.

Altogether six (*Actinobacteria*, *Bacteroidota*, *Bdellovibrionota*, *Campylobacterota*, *Proteobacteria* and *Verrucomicrobiota*) and eight (*Acidobacteriota*, *Bacteroidota*, *Cyanobacteria*, *Firmicutes*, *Nitrospirota*, *Patescibacteria*, *Proteobacteria* and *Verrucomicrobiota*) bacterial phyla had a mean relative abundance ≥1% at least on one sampling occasion in the water and epilithon samples, respectively. The mean relative abundance of taxa *Armatimonadota*, *Chloroflexi*, *Deinococcota*, *Desulfobacterota*, *Fusobacteriota*, *Gemmatimonadota*, *Myxococcota* and NB1-j did not reach 1% at any of the sampling times and points or sample types. Sequences related to phylum *Proteobacteria* and *Bacteriodota* were the most abundant in both water and epilithon samples, comprising up to 41.6–78.6 and 9.8–29.9% of all sequences respectively; followed by representatives of the phylum *Actinobacteria* (11.3% - 29.8%) in the water and phylum *Cyanobacteria* (1.2% - 10.5%) in the epilithon.

**Fig 3:**
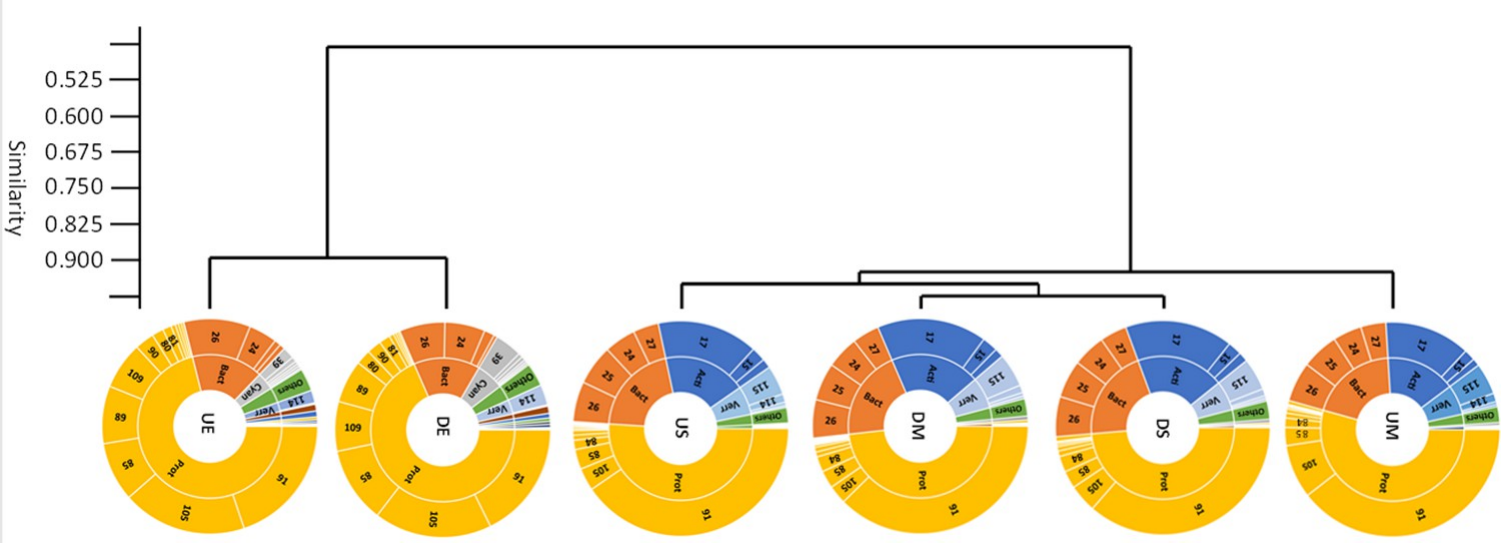
UPGMA dendogram for water and epilithon samples based on the relative abundance ≥0.1% of bacterial orders and pie charts of the given sample types presenting the percentage distribution of relative abundance of bacterial phyla and orders with a mean relative abundance ≥1%. and ≥0.1%, respectively. Letters represent samples taken upstream (U) and downstream (D), from midstream water (M), littoral water (S) and from the pebbles (E). Phyla abbreviations are: Prot—*Proteobacteria*; Bact—*Bacteroidota*; Acti—*Actinobacteriota*; Verr—*Verrucomicrobiota*; Cyan—*Cyanobacteria*. The taxonomic names of orders appear as numbers are listed in S2 Table.

Based on bacterial orders with a mean relative abundance of ≥0.1%, planktonic and epilithic samples also clearly separated (Fig 3). On order level, *Burkholderiales* (*Gammaproteobacteria*) was the most abundant, accounting for more than one-third (mean 38.5%) of all sequences in the water samples. *Frankiales* (*Alphaproteobacteria*) was the second most abundant order (mean 15.2%). Four orders of the phylum *Bacteroidota* (*Chitinophagales*, *Cytophagales*, *Flavobacteriales* and *Sphingobacteriales*) accounted for approximately one-fifth of sequences from water samples. Epilithon samples were dominated by four orders of *Gammaproteobacteria* (*Burkholderiales*, *Pseudomonadales*, *Aeromonadales* and *Xanthomonadales*, representing on average 18.8%, 18.2%, 7.5% and 7.3%, respectively). Other abundant orders in epilithon included *Sphingomonadales* (*Alphaproteobacteria*) and two orders of *Bacteroidota* (*Chitinophagales* and *Flavobacteriales*).

Of the genera identified (S2 Table), *Limnohabitans* (*Comamonadaceae*), *Polynucleobacter* (*Burkholderiaceae*), *Sediminibacterium* (*Chitinophagaceae*), *Fluviicola* (Crocinitomicaceae), *Candidatus_Methylopumilus* (Methylophilaceae), and *Polaromonas* (Comamonadaceae) were present in water samples, while *Acinetobacter* (*Moraxellaceae*), *Aeromonas* (*Aeromonadaceae*), *Arenimonas* (*Xanthomonadaceae*), *Nitrospira*, *Rheinheimera* (Alteromonadaceae), and *Luteolibacter* (*Rubritaleaceae*) occurred in the epilithon. Genera *Flavobacterium* (*Flavobacteriaceae*), *Pseudomonas* (*Pseudomonadaceae*), *Sphingorhabdus* (Sphingomonadaceae), and *Rhodoferax* (*Comamonadaceae*) were identified in both water and epilithon samples.

### Temporal differences in the bacterial communities

**Fig 4:**
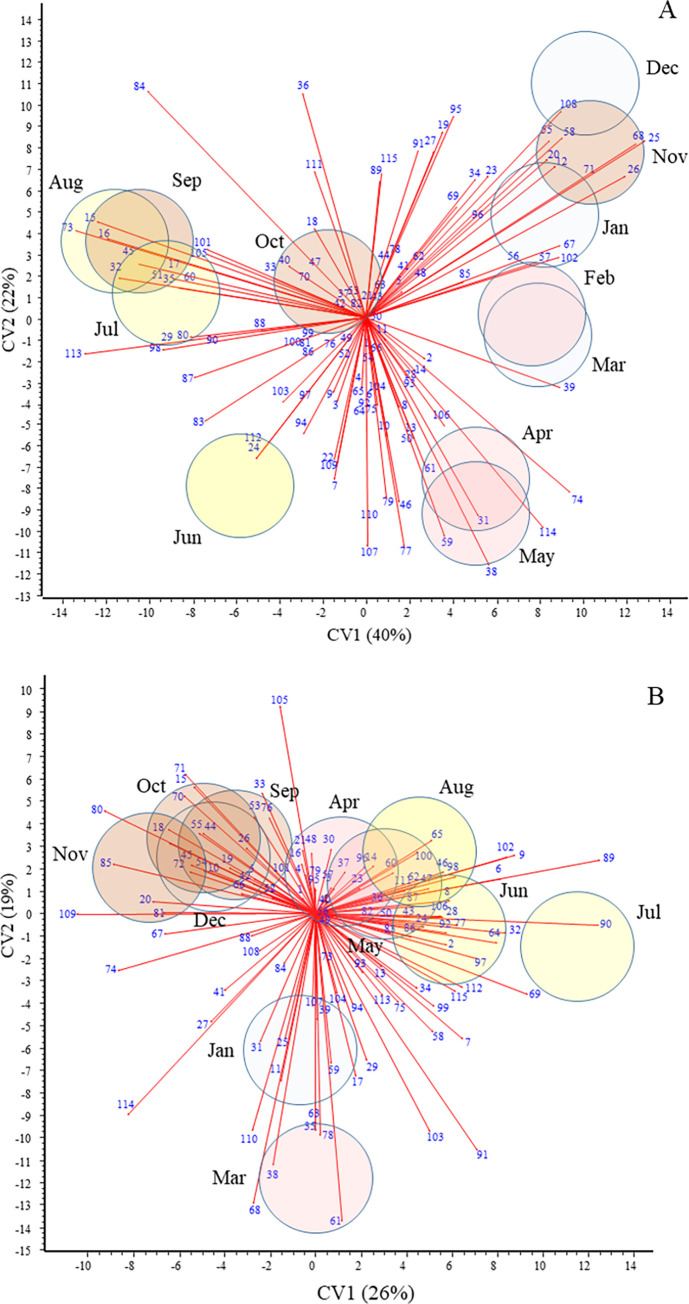
CVA biplots with 95% isodensity circles based on relative abundance of order-level OTUs in water (A) and epilithon (B) samples. Isodensity circles refer to the month of sampling, of which June, July and August, i.e. the‘traditional’ summer months, are filled in yellow. Order-level OTUs are shown as numbered vectors; taxonomic names are listed in S2 Table.

The composition of bacterial communities showed clear temporal separation in both habitats and, similar to physical and chemical parameters of the water, these separations did not coincide completely with the four ‘traditional’ calendar seasons (Fig 4, S2 Table). In water (Fig 4A), unique dominant taxa characterized the bacterial communities in June (e.g. *Chitinophagales*, *Opitutales*), from July to September (e.g. *Bdellovibrionales*, *Microtrichales*, *Synechococcales*), November to January (e.g. *Cytophagales*, *Flavobacteriales*, *Gracilibacteria*, *Saccharimonadales*, *Thiotrichales*), February to March (*Cyanobacteriales*) and April to May (e.g. *Caulobacterales*, *Gemmatimonadales*, *Verrucomicrobiales*). Temporal differences were lower in epilithon than in water and only three main periods were distinguished in this habitat (Fig 4B). These periods were dominated, for instance, by *Gracilibacteria* (January-March), *Aeromonadales*, *Alteromonadales* and *Methylococcales* (April-August) and *Rhizobiales*, *Rhodobacterales*, *Sphingomonadales* (September-December).

**Fig 5:**
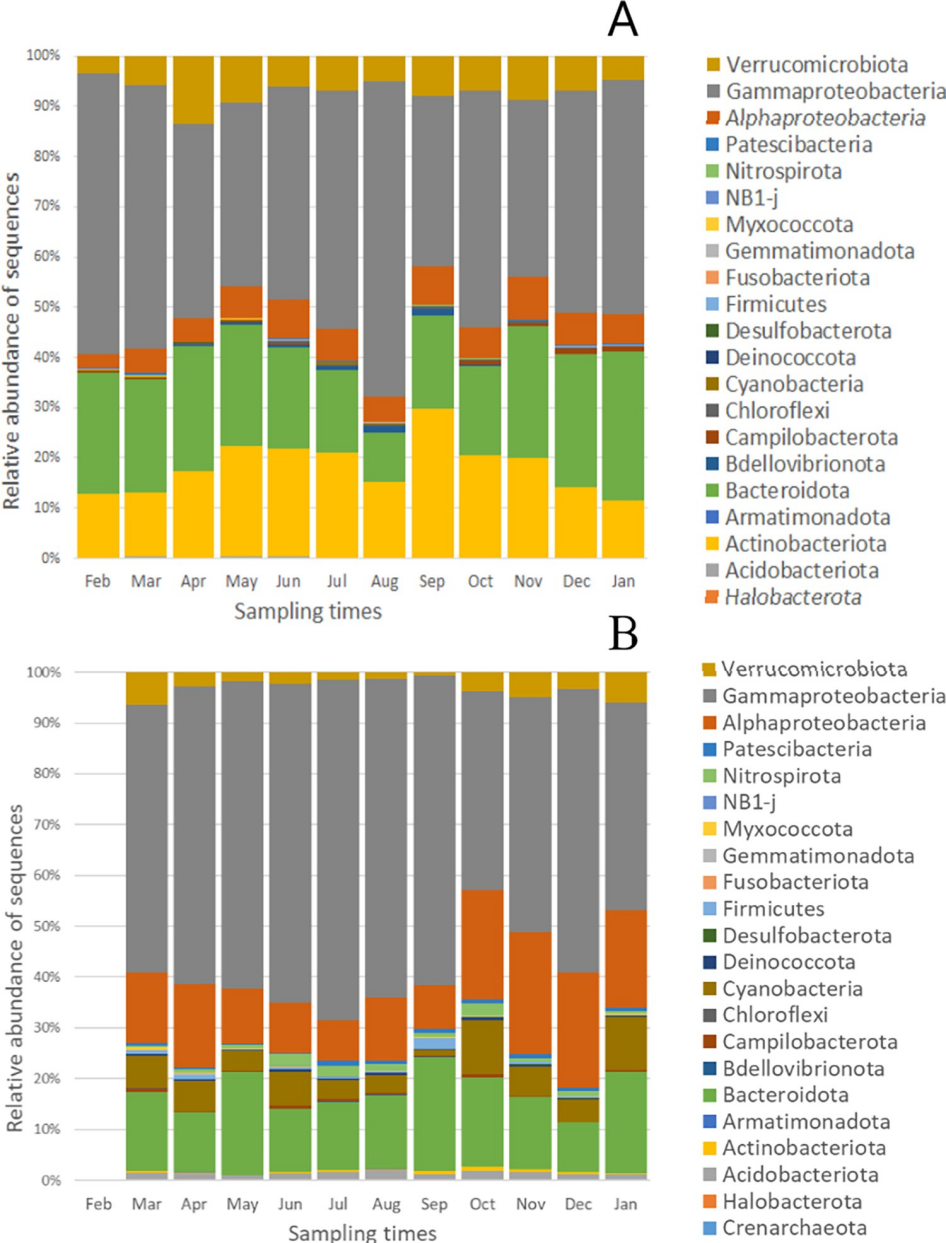
Monthly relative abundance of 16S rRNA gene amplicon sequences at phylum level identified from the Danube water (A) and epilithon (B) samples in the one-year period.

Changes in the relative abundances of bacterial phyla present in ≥1% over time are shown in Fig 5. In the water samples (Fig 5A), phylum *Bacteroidota* showed an increase from October to January and a decrease from May to August. Planktonic *Proteobacteria* were most abundant in August. There was also a late summer maximum for planktonic *Actinobacteria* in September. In the epilithon samples (Fig 5B), the relative abundance values of *Proteobacteria* decreased from July to October and increased from October to December. The relative abundance of sequences related to phyla *Bacteroidota* and *Cyanobacteria* fluctuated during the year in the epilithon. The annual dynamics in the relative abundance of phylum *Alphaproteobacteria* was opposite in the two habitats; reaching the lowest values in water and the highest in epilithon in the coldest months.

### Correlation of key environmental factors and the taxonomic composition of planktonic and epilithon bacterial communities throughout a year

**Fig 6:**
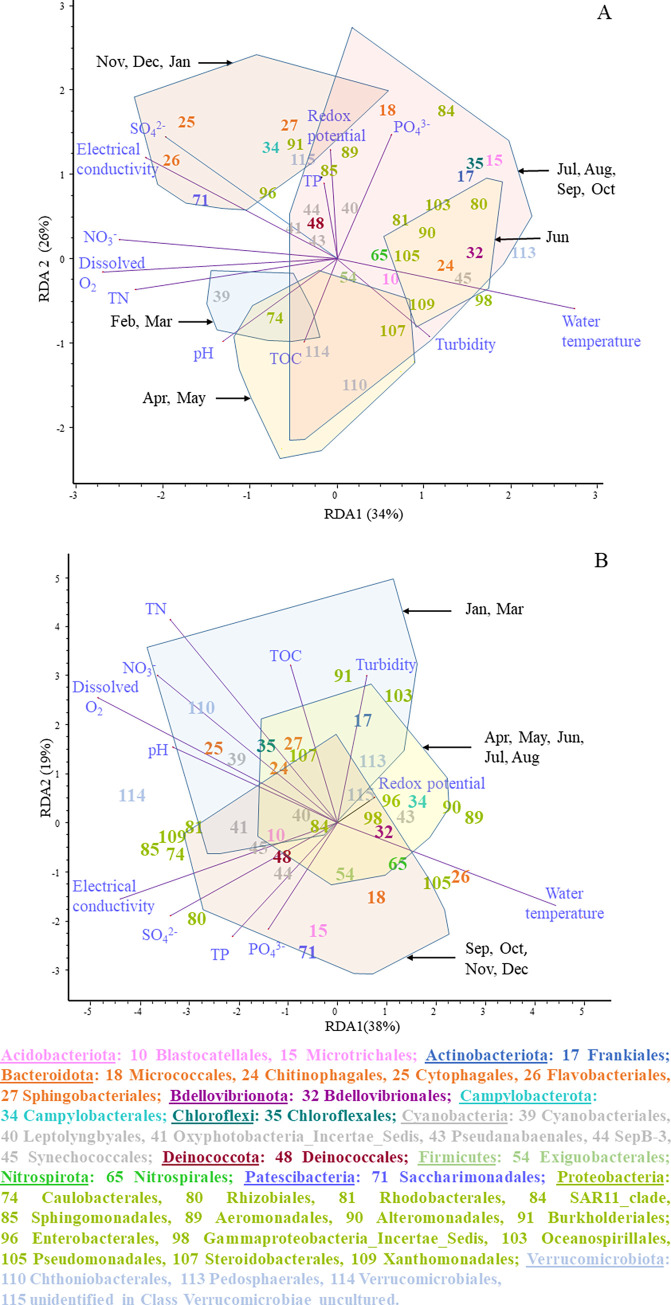
RDA ordinations of water (A) and epilithon (B) samples, based on physical, chemical and microbiological variables. Convex polygons represent samples collected in months grouped by CVAs. Bacterial orders with a mean relative abundance ≥0.1% are shown by numbers and listed at the bottom. Phyla are marked with different colours.

Correlations between environmental characteristics and the composition of bacterial communities can be explored by the joint evaluation of Figs 1, 4 and S2 Table. The first shows physical and chemical variables that affect water quality in different months and the last two provide the main prokaryotic taxa occurring in the habitats at the same time periods. Fig 6 demonstrates these correlations with a single multivariate analysis of both environmental variables and bacterial orders present in ≥0.1% abundance. In the plankton (Fig 6A), *Caulobacterales* and *Verrucomicrobiales*, among others, occurred in the highest proportion when the highest TOC and turbidity, and the lowest phosphate concentration characterized the water (i.e. in April and May). Of the abundant taxa, *Bdellovibrionales*, *Chitinophagales*, *Frankiales*, *Microtrichales*, *Oceanospirillales*, *Pedosphaerales*, *Synechococcales* and *Xanthomonadales* dominated the water during the warmest months. When low water temperatures were coupled with high electric conductivity and high concentrations of sulphate, nitrate, total nitrogen and dissolved oxygen (from November to March), *Burkholderiales*, *Sphingobacteriales*, *Sphingomonadales*, *Cytophagales*, *Flavobacteriales*, *Saccharimonadales* were the most characteristic bacterial orders.

In the epilithon, the dominance of *Aeromonadales* and *Alteromonadales* was observed in the warmest months (Fig 6B). Abundances of *Burkholderiales*, *Chthoniobacterales* and *Cytophyagales*, among others, were strongly associated with the lowest temperature and the highest pH, dissolved oxygen, total nitrogen, TOC and nitrate concentration observed between January and March. From September to December, when electrical conductivity and the concentrations of sulphate, phosphate and total phosphorus peaked, *Rhizobiales*, *Sphingomonadales* and *Saccharimonadales*, for instance, were the most characteristic bacterial orders on pebbles.

## Discussion

This study provides the first, detailed insight into the composition and the spatial and year-round temporal changes of planktonic and epilithic prokaryotic communities of Danube. High-throughput next generation amplicon sequencing was used with OTU-based calculations applying filters to retain clusters with >0.1% relative abundance, enabling a consistent taxonomy comparison [39–42].

River microbiota is mostly studied in water or, less frequently, from sediment and very little is known about epilithon. Differences in microbial community structure in the two former habitats have been reported from the Yellow River estuary [28], the Ibrahim River, Lebanon [26], the sub-arctic Pasvik River, Norway [16], and from the Loa River, Atacama Desert, Chile [43], suggesting that type of river habitat is a key driver in community composition, regardless of climatic conditions. Our study demonstrated strong separation of water and epilithon communities in different Danube sections. Prokaryotic diversity and variance were higher in the epilithon than in the water samples. Both water and epilithon samples were dominated by members of phyla *Proteobacteria* (mostly by *Gammaproteobacteria*, including the former class of *Betaproteobacteria*) and *Bacteroidota*. Sequences related to the latter are regularly detected in high abundance in urban rivers, often explained by anthropogenic origin [27]. In our study, the abundance of *Bacteroidota* was on average three times higher in epilithon than in water. *Cyanobacteria* were also more abundant in the epilithon, while the proportion of sequences related to phylum *Actinobacteria* was higher in the water samples. The number of *Verrucomicrobiota* sequences was also significant in the Danube samples, twice as high in water as in epiliton samples. This picture is consistent with the results of microbial diversity studies in streams [12, 44]. A previous longitudinal study on Danube microbial communities also indicated the dominance of these phyla [45].

Temporal variability in the structure of microbiota has been regularly reported [18, 27, 46–48], however, most investigations focus on one or two seasons, which is insufficient to thoroughly explore seasonality of microbiota reacting to changing environmental conditions throughout the year, especially when seasons themselves are becoming irregular, varying in length and temperature due to climate change. Our year-round examinations demonstrated that summer became longer; temperature and other physico-chemical characteristics of Danube in September were closer to that observed in the summer. This is consistent with the long-term trends related to climate change [2]. Temporal changes in plankton and epilithon microbiota also did not coincide completely with the four equal-length calendar seasons. Based on microbial composition, five main periods were distinguished in the plankton, of which the months from July to September dominated by the same bacterial taxa were good indicators of the protracted summer. Only three main periods were observed in epilithon and this community appeared less responsive to the shift of seasons related to climate change than the plankton. Similar result was obtained by Liu et al. [25], observing significantly higher seasonal variation in planktonic bacterial communities of the Yangtze River than in its sediments. Laboratory simulation indicated that epilithic communities react more strongly to hydrological stress than to warming [49]. Temperature was found to be the most important environmental factor influencing the seasonal composition of bacterial communities in the Danube, as in the case of other rivers [20, 21, 50, 51]. Among the investigated physico-chemical parameters, previous studies in rivers have mainly highlighted the impact of nitrogen forms (e.g. nitrate, TN, DIN) on the bacterial community structure, especially in sediment samples [52–56]. In our study, abundance of bacteria in the Danube was negatively correlated both with nitrogen forms and dissolved oxygen in water and sediment samples.

Main taxa depending on temperature were also determined; water temperature correlated positively with *Gammaproteobacteria* in plankton and negatively with *Alphaproteobacteria* in epilithon samples. The phyla *Actinobacteria* and *Bacteroidota* have shown opposite temporal dynamics in Danube water: the relative abundance of *Actinobacteria* was the highest in the warmest while *Bacteroidota* in the coldest months. Higher water temperatures were reflected in the appearance of phototrophic (e.g. *Synechococcales*) and aerobic and facultative anaerobic heterotrophic (e.g. *Alteromonadales*, *Chitinophagales*, *Pseudomonadales*, *Rhizobiales*, *Xanthomonadales*) bacterial taxa. The higher abundance of sequences related to *Cytophagales* and *Flavobacteriales* (*Bacteroidota*) in the cold period can be connected to the biodegradation of algal organic matter, as it was demonstrated earlier [57].

Comparing planktonic bacterial communities in the midstream of the river and near the shore, similar taxonomic composition was observed at different distances of the shore, which indicates high waving and stirring effect in the studied sections of the Danube.

To explore the possible impact of a large city on the microbiota of the Danube, samples taken both upstream and downstream from the Hungarian capital were also investigated, but no significant differences were observed between the microbial communities of the two river sections. Previous reports demonstrated substantial and sometimes dramatic effects of domestic sewage input and excessive human use on river microbiota, e.g. in the Qingliu River, China [15] and in the urban sites of River Ganges, India [58]. But this observation is not universal: studying the microbiome of the River Nile, [24] found a “striking stability” of community structure in Cairo metropolitan areas and [27] also reported a lack of significant spatial differences in the bacterioplankton along an urbanization gradient of the Ganjiang River, China. In the Danube, a potential reason for the homogeneity in community structure upstream and downstream of Budapest is the lack of untreated sewage input: since the installation of the central wastewater treatment plant in 2010, the emission of untreated wastewater is negligible in the Hungarian capital [59]. There were no designated bathing sites at the time of sampling and other recreational use was limited. Other urban anthropogenic impact, such as run-off contamination from paved surfaces is probably counteracted by the large flow volume of the river (45–85 km^3^/year), which results in a significant dilution of external contamination.

Many taxa identified in this study have also been found in other rivers, and their role in the ecosystems and potential effects on human health are also known in some cases. *Limnohabitans*, *Polynucleobacter*, hgcI_clade and *Sediminibacterium* were recorded in rivers worldwide [16, 18, 60–65]. The genus *Limnohabitans* includes morphologically diverse, metabolically versatile, fast-growing bacteria which play an important role in channelling carbon from primary producers to higher trophic levels [66]. Members of *Sediminibacterium* are strictly aerobic chemoorganotrophic bacteria which are capable of growth both free-living and in biofilm depending on redox conditions and nutrient supply of the environment [67]. Other genera characteristic of both plankton and epilithon (e.g. *Polaromonas*, *Pseudomonas* and *Flavobacterium*) can play key roles in the carbon and nitrogen cycles by decomposition of various organic compounds, including pollutants and/or xenobiotics [68, 69]. Genera *Achromobacter*, *Comamonas*, *Dechloromonas* and *Malikia* were shown earlier to be very efficient in degrading chlorinated or aromatic micropollutants [70–73], and *Dechloromonas*, *Flavobacterium* and *Polaromonas* were also associated previously with the transformation or accumulation of heavy metals [68, 74]. Some taxa demonstrated in the Danube epilithon samples may harbour potential pathogens (e.g. *Aeromonas*, *Acinetobacter*, *Pseudomonas*, *Legionella*) however, the abundance of these genera does not indicate direct risk to human health either via recreational use or drinking water production. Two genera of the phylum *Bacteriodota*, *Flavobacterium* and *Pedobacter*, which were abundant in the coldest months in epilithon and water samples respectively, are known to harbour intrinsic antimicrobial resistance mechanism and they were recovered as dominant genera in a study on antimicrobial resistant organisms in drinking water supply [75]. Other detected genera (e.g *Aetherobacter* and *Acinetobacter*) also harbour chromosomal or plasmid-borne resistance to various antibiotics [76, 77].

## Conclusions

High-frequency sampling is necessary year-round to reveal how microbiota reflects the changing environment during climate change, when the seasons are irregular. In rivers, climate change may result in a long-term, year-on-year warming and, as our study also demonstrated, a seasonal increase in water temperature due to the prolonged summers.

River habitats predominantly determine the taxonomic composition of the microbiota; diverse and well-differentiated microbial communities developed in water and epilithon. Bacterial diversity was greater in epilithon than in plankton, and the latter, due to waving and stirring of the river, hosted homogenous communities in the midstream and near the shore. Probably due to the lack of untreated sewage input and the dilution of external contamination, the large city in the study area had no significant effect on the bacterial communities.

The temporal variability of the microbiota reflected the changing climatic conditions differently in the two investigated habitats. Bacterial composition of plankton was reflecting the prolongation of the summer, while epilithon appeared less responsive to the changes. The main taxa indicating rising water temperature could be identified, as well as the relationships between several further environmental factors (such as turbidity, TOC, electric conductivity, pH and the concentration of phosphate, sulphate, nitrate, total nitrogen and the dissolved oxygen) and the composition of the microbiota in a large river.

Shift in microbial community responding to changing environment may be of crucial ecological and human health consequences.

## Supporting information

S1 Fig Alfa diversity: The rarefaction curves of the observed features (numbers of OTUs found) in each habitat. (TIF)

S2 Fig Beta diversity: PCoA of the weighted unifrac distances of the samples. (TIF)

S1 Table Monthly physical and chemical parameters (average ± SD) of the water taken upstream (U) and downstream (D), from midstream (M) and the littoral zone (S). Different letters indicate significant statistical difference (p < 0.05).
(DOCX)

S2 Table Order-level OTUs represented by numbers in Figs 2 and 4, their dominant occurrence in water and epilithon, number of reads, and relevant genera and species assigned to the OTUs. (DOCX)
