# Supplementary figures and images for: Planktonic and epilithic prokaryota community compositions in a large temperate river reflect climate change related seasonal shifts
Source: PLoS One. 2023 Sep 21;18(9):e0292057. doi: 10.1371/journal.pone.0292057 (PMC10513243; doi:10.1371/journal.pone.0292057)

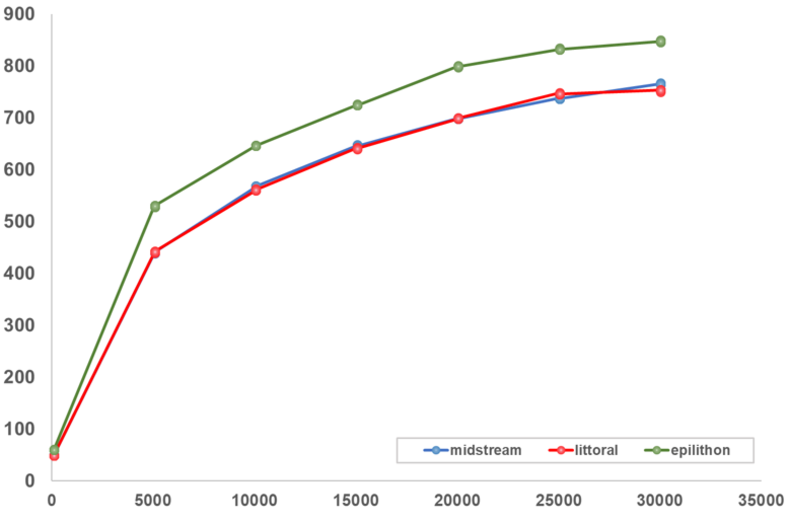

Supplement: S1 Fig — (TIF) [file pone.0292057.s001.tif]

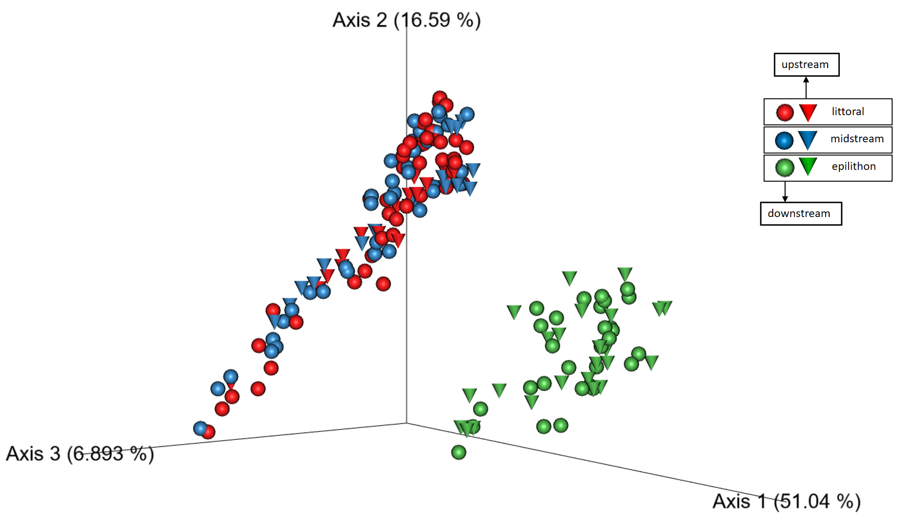

Supplement: S2 Fig — (TIF) [file pone.0292057.s002.tif]
