# Supplementary material for: Planktonic and epilithic prokaryota community compositions in a large temperate river reflect climate change related seasonal shifts
Source: PLoS One. 2023 Sep 21;18(9):e0292057. doi: 10.1371/journal.pone.0292057 (PMC10513243; doi:10.1371/journal.pone.0292057)
Supplement: S1 Table — Different letters indicate significant statistical difference (p < 0.05). (DOCX) [file pone.0292057.s003.docx]

**S1 Table.**

|  | **Water temperature (°C)** | | | | **Electrical conductivity (mS)** | | | | **pH** | | | | **Turbidity (ntu)** | | | | **Dissolved oxygen (mg/l)** | | | | **Redox potential (mV)** | | | | |
| --- | --- | --- | --- | --- | --- | --- | --- | --- | --- | --- | --- | --- | --- | --- | --- | --- | --- | --- | --- | --- | --- | --- | --- | --- | --- |
| **Month** | **UM** | **US** | **DM** | **DS** | **UM** | **US** | **DM** | **DS** | **UM** | **US** | **DM** | **DS** | **UM** | **US** | **DM** | **DS** | **UM** | **US** | **DM** | **DS** | **UM** | **US** | **DM** | **DS** |  |
| **1** | 1.83  ±0.27  a | 1.83  ±0.19  a | 1.58  ±0.13  ab | 1.30  ±0.20  b | 0.38  ±0.02  a | 0.40  ±0.01  a | 0.40  ±0.01  a | 0.40  ±0.01  a | 7.82  ±0.15  a | 7.84  ±0.14  a | 8.01  ±0.13  a | 8.06  ±0.22  a | 6.70  ±1.59  a | 7.04  ±2.96  a | 7.05  ±2.44  a | 8.76  ±3.31  a | 11.85  ±0.18  a | 11.68  ±0.35  a | 11.87  ±0.41  a | 11.92  ±0.44  a | 100.97  ±3.72  a | 100.20  ±3.53  a | 101.87  ±3.96  a | 102.25  ±2.85  a |  |
| **2** | 3.80  ±0.82  a | 4.17  ±0.55  a | 4.27  ±0.31  a | 4.27  ±0.59  a | 0.43  ±0.01  a | 0.42  ±0.01  a | 0.42  ±0.01  a | 0.43  ±0.01  a | 7.73  ±0.22  a | 7.79  ±0.09  ab | 8.07  ±0.12  ab | 8.10  ±0.11  b | 7.33  ±0.27  a | 7.89  ±0.84  a | 4.69  ±0.39  b | 4.77  ±0.75  b | 11.67  ±0.31  a | 11.80  ±0.53  a | 12.17  ±0.32  a | 11.60  ±0.26  a | 137.33  ±5.03  a | 126.33  ±9.71  a | 142.33  ±13.43  a | 137.33  ±35.35  a |  |
| **3** | 6.27  ±0.23  a | 6.38  ±0.17  a | 6.18  ±0.15  a | 6.33  ±0.18  a | 0.33  ±0.04  a | 0.34  ±0.02  a | 0.35  ±0.02  a | 0.35  ±0.02  a | 8.09  ±0.41  a | 7.90  ±0.09  a | 8.09  ±0.06  a | 8.06  ±0.03  a | 72.02  ±54.82  a | 53.50  ±44.26  a | 47.85  ±24.97  a | 37.05  ±21.51  a | 11.17  ±0.15  a | 11.06  ±0.18  a | 11.24  ±0.49  a | 11.25  ±0.20  a | 148.97  ±113.40  a | 155.12  ±117.09  a | 118.60  ±92.23  a | 99.20  ±77.96  a |  |
| **4** | 11.01  ±1.60  a | 11.46  ±1.55  a | 11.23  ±1.38  a | 11.58  ±1.27  a | 0.32  ±0.01  a | 0.32  ±0.01  a | 0.32  ±0.01  a | 0.32  ±0.01  a | 7.96  ±0.19  a | 7.92  ±0.12  a | 8.05  ±0.08  a | 8.05  ±0.12  a | 14.10  ±4.97  a | 26.04  ±13.96  b | 15.79  ±5.05  ab | 18.20  ±4.17  ab | 9.77  ±0.16  a | 9.64  ±0.32  a | 10.36  ±0.22  b | 10.38  ±0.26  b | 42.79  ±24.38  a | 43.97  ±28.26  a | 28.57  ±15.57  a | 28.72  ±16.65  a |  |
| **5** | 13.40  ±0.56  a | 13.73  ±0.29  a | 13.07  ±1.14  a | 13.05  ±1.26  a | 0.29  ±0.01  a | 0.29  ±0.01  a | 0.30  ±0.01  a | 0.29  ±0.02  a | 7.54  ±0.18  a | 7.53  ±0.17  a | 7.81  ±0.19  a | 7.81  ±0.22  a | 75.83  ±66.67  a | 49.72  ±40.39  a | 56.70  ±46.03  a | 53.67  ±43.22  a | 9.98  ±0.15  a | 9.50  ±0.64  a | 10.10  ±0.73  a | 9.93  ±0.88  a | 77.95  ±75.58  a | 72.58  ±90.45  a | 90.62  ±51.79  a | 69.38  ±49.29  a |  |
| **6** | 19.07  ±0.37  a | 19.48  ±0.72  ab | 19.95  ±0.36  bc | 20.48  ±0.21  c | 0.26  ±0.00  a | 0.26  ±0.00  a | 0.27  ±0.01  a | 0.26  ±0.00  a | 7.52  ±0.14  a | 7.53  ±0.25  a | 7.55  ±0.26  a | 7.56  ±0.22  a | 83.08  ±10.87  a | 64.42  ±12.53  ab | 51.33  ±7.05  bc | 43.32  ±15.50  c | 8.30  ±0.22  a | 7.85  ±0.34  a | 8.33  ±0.36  a | 8.08  ±0.43  a | 97.22  ±26.44  a | 98.37  ±28.32  a | 91.55  ±15.88  a | 92.65  ±21.15  a |  |
| **7** | 21.20  ±0.53  a | 21.72  ±0.57  ab | 21.70  ±0.28  ab | 22.13  ±0.51  b | 0.27  ±0.02  a | 0.27  ±0.02  a | 0.28  ±0.02  a | 0.28  ±0.02  a | 7.42  ±0.18  a | 7.35  ±0.10  a | 7.49  ±0.16  a | 7.50  ±0.19  a | 15.18  ±4.29  a | 48.63  ±41.86  a | 19.97  ±1.83  a | 18.78  ±3.14  a | 8.47  ±0.30  a | 8.08  ±0.39  a | 8.70  ±0.54  a | 8.53  ±0.41  a | 54.25  ±1.76  a | 61.37  ±6.74  b | 57.72  ±4.34  ab | 57.45  ±1.67  ab |  |
| **8** | 21.73  ±0.50  a | 22.35  ±0.84  a | 21.78  ±0.18  a | 22.10  ±0.17  a | 0.29  ±0.02  a | 0.29  ±0.02  a | 0.30  ±0.02  a | 0.30  ±0.02  a | 7.59  ±0.15  ab | 7.49  ±0.09  b | 7.65  ±0.03  a | 7.65  ±0.06  a | 18.92  ±6.95  a | 21.85  ±7.39  a | 18.23  ±2.87  a | 18.13  ±3.75  a | 8.15  ±0.24  ab | 7.97  ±0.31  a | 8.50  ±0.09  c | 8.35  ±0.10  bc | 64.32  ±21.22  a | 61.55  ±9.86  a | 91.47  ±21.07  a | 90.55  ±20.75  a |  |
| **9** | 18.69  ±2.84  a | 19.04  ±3.12  a | 18.56  ±2.41  a | 18.52  ±2.25  a | 0.32  ±0.01  a | 0.32  ±0.01  a | 0.33  ±0.02  a | 0.33  ±0.01  a | 7.50  ±0.37  a | 7.54  ±0.35  a | 7.44  ±0.21  a | 7.49  ±0.19  a | 12.67  ±1.98  a | 22.48  ±14.26  b | 12.72  ±4.11  ab | 12.86  ±3.09  ab | 8.83  ±0.37  a | 8.58  ±0.40  a | 8.72  ±0.35  a | 8.41  ±0.32  a | 120.37  ±24.34  a | 123.29  ±28.73  a | 124.34  ±15.87  a | 123.97  ±14.96  a |  |
| **10** | 12.92  ±0.08  a | 12.98  ±0.19  a | 12.55  ±0.53  a | 12.57  ±0.55  a | 0.35  ±0.01  a | 0.34  ±0.01  a | 0.35  ±0.01  a | 0.36  ±0.02  a | 7.16  ±0.31  a | 7.27  ±0.23  a | 7.47  ±0.19  a | 7.46  ±0.27  a | 10.84  ±5.08  a | 13.65  ±7.76  a | 15.76  ±14.84  a | 13.28  ±7.43  a | 9.77  ±0.16  a | 9.58  ±0.26  a | 9.70  ±0.15  a | 9.57  ±0.19  a | 138.67  ±25.43  a | 135.77  ±25.09  a | 99.57  ±31.93  a | 100.90  ±33.33  a |  |
| **11** | 8.22  ±1.99  a | 8.22  ±2.06  a | 8.17  ±1.57  a | 8.27  ±1.58  a | 0.39  ±0.04  a | 0.39  ±0.04  a | 0.39  ±0.05  a | 0.39  ±0.05  a | 7.74  ±0.65  a | 7.82  ±0.57  a | 8.09  ±0.26  a | 8.11  ±0.26  a | 12.24  ±5.44  a | 13.19  ±7.52  a | 20.90  ±25.22  a | 11.00  ±5.43  a | 9.97  ±0.15  a | 9.92  ±0.20  ab | 9.73  ±0.20  ab | 9.57  ±0.29  b | 98.97  ±5.60  a | 98.97  ±4.30  a | 110.77  ±4.27  b | 117.93  ±5.83  b |  |
| **12** | 3.40  ±0.22  a | 3.48  ±0.19  a | 3.98  ±0.15  b | 3.97  ±0.15  b | 0.38  ±0.01  a | 0.38  ±0.01  a | 0.38  ±0.01  a | 0.38  ±0.02  a | 7.34  ±0.76  a | 7.41  ±0.58  a | 7.88  ±0.19  a | 7.92  ±0.21  a | 8.68  ±3.62  a | 8.94  ±4.86  a | 11.38  ±3.62  a | 11.38  ±2.63  a | 10.80  ±0.21  a | 10.77  ±0.18  a | 10.75  ±0.14  a | 10.73  ±0.15  a | 92.40  ±2.60  a | 92.58  ±2.52  a | 94.42  ±0.69  a | 96.55  ±5.99  a |  |

|  | **TOC (mg/L)** | | | | **TN (mg/L)** | | | | **TP (μg/L)** | | | | **NO_3_^-^ (mg/L)** | | | | **PO_4_^3-^ (μg/L)** | | | | **SO_4_^2-^ (mg/L)** | | | |
| --- | --- | --- | --- | --- | --- | --- | --- | --- | --- | --- | --- | --- | --- | --- | --- | --- | --- | --- | --- | --- | --- | --- | --- | --- |
| **Month** | **UM** | **US** | **DM** | **DS** | **UM** | **US** | **DM** | **DS** | **UM** | **US** | **DM** | **DS** | **UM** | **US** | **DM** | **DS** | **UM** | **US** | **DM** | **DS** | **UM** | **US** | **DM** | **DS** |
| **1** | 1.74  ±0.32  a | 1.90  ±0.16  a | 1.61  ±0.22  a | 1.57  ±0.26  a | 2.29  ±0.04  a | 2.29  ±0.02  a | 2.43  ±0.06  b | 2.40  ±0.03  b | 120.05  ±22.82  a | 104.83  ±34.44  a | 136.01  ±16.82  a | 141.25  ±27.50  a | 10.12  ±0.04  a | 10.18  ±0.05  a | 10.24  ±0.14  a | 10.14  ±0.02  a | 128.33  ±21.37  ac | 118.33  ±13.29  a | 161.67  ±9.83  b | 146.67  ±19.66  bc | 35.16  ±1.77  a | 35.36  ±1.54  a | 35.98  ±1.67  a | 36.62  ±1.95  a |
| **2** | 2.24  ±0.56  a | 1.75  ±0.24  a | 1.89  ±0.15  a | 1.67  ±0.08  a | 2.94  ±0.03  a | 2.90  ±0.04  a | 3.07  ±0.03  b | 3.18  ±0.02  c | 156.97  ±16.03  a | 284.24  ±134.98  a | 215.56  ±88.31  a | 142.83  ±21.28  a | 11.04  ±0.05  a | 11.00  ±0.05  a | 11.16  ±0.04  b | 11.02  ±0.03  a | 90.00  ±0.00  a | 103.33  ±15.28  a | 136.67  ±35.12  a | 100.67  ±16.77  a | 35.60  ±0.13  a | 36.24  ±0.34  b | 36.70  ±0.18  bc | 36.93  ±0.07  c |
| **3** | 2.95  ±0.90  a | 2.19  ±0.53  a | 2.90  ±0.83  a | 3.26  ±0.89  a | 2.76  ±0.21  a | 2.68  ±0.24  a | 2.70  ±0.21  a | 2.66  ±0.21  a | 125.66  ±37.44  a | 135.76  ±31.78  a | 127.68  ±48.70  a | 149.90  ±54.26  a | 9.94  ±0.79  a | 10.09  ±0.75  a | 9.96  ±0.81  a | 9.84  ±0.80  a | 116.67  ±36.15  a | 106.67  ±40.33  a | 128.33  ±24.01  a | 145.00  ±49.70  a | 26.27  ±2.73  a | 26.15  ±2.72  a | 26.22  ±3.09  a | 26.59  ±3.16  a |
| **4** | 1.58  ±0.15  ac | 2.87  ±0.91  b | 1.79  ±0.10  ac | 2.02  ±0.41  a | 1.85  ±0.33  a | 1.97  ±0.32  a | 1.91  ±0.36  a | 1.91  ±0.33  a | 64.66  ±22.00  a | 80.25  ±17.88  a | 105.27  ±41.66  a | 113.25  ±64.96  a | 6.96  ±1.40  a | 7.04  ±1.54  a | 6.99  ±1.39  a | 6.91  ±1.28  a | 52.22  ±25.39  a | 55.56  ±23.51  a | 80.00  ±47.96  a | 93.33  ±66.90  a | 25.45  ±1.00  a | 25.50  ±1.05  a | 26.11  ±1.00  a | 26.45  ±0.97  a |
| **5** | 4.14  ±1.67  a | 2.55  ±0.88  a | 2.65  ±1.14  a | 2.69  ±0.92  a | 1.86  ±0.43  a | 1.72  ±0.37  a | 1.79  ±0.36  a | 1.84  ±0.42  a | 55.38  ±22.69  a | 55.02  ±35.77  a | 76.44  ±43.89  a | 78.08  ±44.61  a | 6.28  ±0.94  a | 6.18  ±0.96  a | 6.26  ±0.93  a | 6.22  ±0.88  a | 106.67  ±62.18  a | 105.00  ±56.83  a | 111.67  ±79.85  a | 110.00  ±75.63  a | 20.59  ±3.02  a | 20.44  ±2.98  a | 21.25  ±2.87  a | 21.45  ±3.10  a |
| **6** | 1.76  ±0.06  a | 1.89  ±0.30  a | 1.89  ±0.16  a | 1.93  ±0.18  a | 1.22  ±0.12  a | 1.26  ±0.17  a | 1.26  ±0.12  a | 1.30  ±0.12  a | 85.84  ±11.50  a | 89.17  ±10.67  a | 88.95  ±8.80  a | 90.98  ±31.80  a | 4.73  ±0.36  a | 4.68  ±0.33  a | 4.84  ±0.42  a | 5.04  ±0.62  a | 143.33  ±23.38  a | 143.33  ±25.82  a | 121.67  ±23.17  a | 141.67  ±14.72  a | 20.16  ±0.45  ab | 19.68  ±0.28  a | 20.37  ±0.57  ab | 20.80  ±0.55  b |
| **7** | 1.47  ±0.10  a | 1.54  ±0.45  a | 1.37  ±0.08  a | 1.47  ±0.12  a | 1.21  ±0.48  a | 1.11  ±0.13  a | 1.09  ±0.01  a | 1.07  ±0.02  a | 131.15  ±61.62  a | 127.00  ±52.17  a | 104.70  ±20.69  a | 78.28  ±26.83  a | 3.78  ±0.18  a | 3.72  ±0.11  a | 3.72  ±0.05  a | 3.67  ±0.14  a | 80.00  ±16.73  a | 123.33  ±42.74  ab | 135.00  ±15.17  b | 145.00  ±27.39  b | 23.08  ±1.72  a | 23.12  ±1.26  a | 23.16  ±2.46  a | 23.40  ±3.07  a |
| **8** | 1.31  ±0.22  a | 1.54  ±0.58  a | 1.27  ±0.14  a | 1.40  ±0.10  a | 1.20  ±0.18  a | 1.20  ±0.19  a | 1.20  ±0.08  a | 1.18  ±0.09  a | 126.31  ±51.13  a | 103.00  ±13.92  a | 112.04  ±42.25  a | 127.43  ±41.07  a | 3.79  ±0.32  a | 3.80  ±0.32  a | 3.95  ±0.35  a | 3.84  ±0.36  a | 128.33  ±42.15  a | 135.00  ±28.81  a | 168.33  ±47.08  a | 178.33  ±61.13  a | 24.89  ±0.62  ab | 24.39  ±0.20  b | 25.46  ±0.71  a | 25.82  ±0.73  a |
| **9** | 1.56  ±0.08  a | 1.73  ±0.24  a | 1.54  ±0.11  a | 1.62  ±0.11  a | 1.30  ±0.12  a | 1.32  ±0.11  a | 1.36  ±0.06  a | 1.36  ±0.05  a | 151.79  ±42.10  a | 146.26  ±34.63  a | 152.92  ±43.64  a | 136.84  ±47.67  a | 4.86  ±0.28  a | 4.81  ±0.30  a | 5.13  ±0.25  a | 5.09  ±0.23  a | 126.67  ±29.58  a | 131.11  ±30.18  a | 156.67  ±15.81  a | 151.11  ±20.88  a | 26.56  ±1.77  a | 26.47  ±1.89  a | 27.85  ±2.29  a | 28.05  ±1.49  a |
| **10** | 1.78  ±0.13  a | 1.69  ±0.29  a | 1.78  ±0.35  a | 1.49  ±0.19  a | 1.55  ±0.03  a | 1.64  ±0.26  a | 1.63  ±0.04  a | 1.62  ±0.08  a | 95.10  ±2.77  a | 83.13  ±4.92  a | 144.19  ±46.06  b | 115.33  ±32.07  ab | 5.86  ±0.09  a | 5.85  ±0.06  a | 5.94  ±0.12  a | 5.98  ±0.17  a | 116.67  ±5.16  a | 113.33  ±5.16  a | 120.00  ±0.00  a | 116.67  ±5.16  a | 28.34  ±0.14  a | 28.20  ±0.10  a | 29.32  ±0.40  b | 29.91  ±1.00  b |
| **11** | 1.77  ±0.20  a | 1.93  ±0.19  a | 1.66  ±0.36  a | 1.52  ±0.36  a | 1.71  ±0.17  a | 1.74  ±0.17  a | 1.80  ±0.11  a | 1.82  ±0.13  a | 150.54  ±35.44  a | 153.91  ±30.21  a | 131.14  ±9.05  a | 132.42  ±13.79  a | 6.45  ±0.45  a | 6.47  ±0.48  a | 6.63  ±0.41  a | 6.67  ±0.41  a | 136.67  ±10.33  ab | 131.67  ±16.02  a | 161.67  ±7.53  bc | 168.33  ±24.01  c | 30.55  ±1.44  a | 30.97  ±1.30  a | 31.19  ±1.43  a | 31.25  ±1.75  a |
| **12** | 1.53  ±0.08  a | 1.65  ±0.16  a | 1.61  ±0.11  a | 1.62  ±0.07  a | 1.86  ±0.15  a | 1.86  ±0.11  a | 1.95  ±0.10  a | 1.90  ±0.12  a | 96.10  ±27.94  a | 111.57  ±26.15  a | 126.59  ±26.45  a | 113.43  ±17.47  a | 9.00  ±0.35  ab | 8.85  ±0.26  a | 9.31  ±0.10  b | 9.20  ±0.26  ab | 135.00  ±10.49  a | 121.67  ±17.22  a | 183.33  ±19.66  b | 176.67  ±18.62  b | 34.17  ±1.18  a | 33.96  ±1.00  a | 35.00  ±1.07  a | 35.64  ±1.17  a |
