# Supplementary material for: Planktonic and epilithic prokaryota community compositions in a large temperate river reflect climate change related seasonal shifts
Source: PLoS One. 2023 Sep 21;18(9):e0292057. doi: 10.1371/journal.pone.0292057 (PMC10513243; doi:10.1371/journal.pone.0292057)
Supplement: S2 Table — (DOCX) [file pone.0292057.s004.docx]

**S2 Table.**

^1^ Presented in Figures 2 and 4.

^2^ Dominant OTUs in water (W) or epilithon (E) samples (Fig 2).

^3^ Dominant OTUs in different months in water samples (Fig 4A).

^4^ Dominant OTUs in different months in epilithon samples (Fig 4B).

| **Phylum** | **Serial number of OTU^1^** | **Order** | **Water or epilithon^2^** | **Water^3^** | | | | | **Epilithon^4^** | | | **No of reads** | **Relevant genera and species**  **(the latter in brackets)** |
| --- | --- | --- | --- | --- | --- | --- | --- | --- | --- | --- | --- | --- | --- |
|  |  |  |  | Months | | | | | Months | | |  |  |
|  |  |  |  | Feb, Mar | Apr, May | Jun | Jul, Aug, Sep, Oct, | Nov, Dec, Jan | Jan, Mar | Apr, May, Jun, Jul, Aug | Sep, Oct, Nov, Dec |  |  |
| Crenarchaeota | 1 | Bathyarchaeia | E |  |  |  |  |  |  |  |  | 1391 | *Bathyarchaeia* |
|  | 2 | Nitrosopumilales | E |  |  |  |  |  |  | + |  | 744 | *Nitrosarchaeum* |
| Halobacterota | 3 | Methanomicrobiales | E |  |  |  |  |  |  |  |  | 1970 | *Methanoregula* |
|  | 4 | Methanosarciniales | E |  |  |  |  |  |  |  |  | 2832 | *Methanosaeta, Methanosarcina* |
|  | 5 | unidentified in Domain Bacteria | E |  |  |  |  |  |  |  | + | 1853 |  |
| Acidobacteriota | 6 | unidentified in Class Acidobacteriae | E |  |  |  |  |  |  | + |  | 3119 |  |
|  | 7 | Acidobacteriae | E |  | + | + |  |  |  | + |  | 1167 | *Paludibaculum* |
|  | 8 | Bryobacterales | E |  |  |  |  |  |  | + |  | 1413 | *Bryobacter* |
|  | 9 | 11-24 | E |  |  |  |  |  |  | + |  | 466 |  |
|  | 10 | Blastocatellales | E |  | + |  |  |  |  |  | + | 20399 | *Aridibacter, Blastocatella, Stenotrophobacter* |
|  | 11 | Elev-16S-573 | E |  |  |  |  |  | + |  |  | 589 |  |
|  | 12 | Holophagales | W |  |  |  |  | + |  |  |  | 601 |  |
|  | 13 | Subgroup_17 | E |  | + |  |  |  |  |  |  | 1345 |  |
|  | 14 | Vicinamibacterales | E |  |  |  |  |  |  | + |  | 6025 |  |
| Actinobacteriota | 15 | Microtrichales | W |  |  |  | + |  |  |  | + | 97458 | *Ilumatobacter* |
|  | 16 | unidentified in Class Actinobacteria | W |  |  |  | + |  |  |  |  | 514 |  |
|  | 17 | Frankiales | W |  |  |  | + |  | + |  |  | 669746 |  |
|  | 18 | Micrococcales | W |  |  |  |  |  |  |  | + | 59969 | *Aurantimicrobium, Rhodoluna* |
|  | 19 | PeM15 | W |  |  |  |  | + |  |  | + | 1883 |  |
|  | 20 | unidentified in  Class Actinobacteria uncultured | W |  |  |  |  | + |  |  | + | 746 |  |
|  | 21 | Gaiellales | E |  |  |  |  |  |  |  |  | 2713 | *Gaiella* |
| Armatimonadota | 22 | Fimbriimonadales | E |  | + | + |  |  |  |  | + | 791 |  |
| Bacteroidota | 23 | Bacteroidales |  |  |  |  |  | + |  |  |  | 4690 | *Bacteroides (B. graminisolvens), Mucinivorans (M. hirudinis)* |
|  | 24 | Chitinophagales | E |  |  | + |  |  |  | + |  | 328988 | *Aurantisolimonas, Dinghuibacter, Edaphobaculum, Ferruginibacter (F. profundus, F. alkalilentus), Flavihumibacter, Lacibacter, Parasegetibacter (P. luojiensis), Rurimicrobium (R. arvi), Sediminibacterium, Terrimonas (T. lutea), Haliscomenobacter, Phaeodactylibacter, Portibacter* |
|  | 25 | Cytophagales | W |  |  |  |  | + | + |  |  | 277988 | *Algoriphagus, Cytophaga, Hymenobacter (H. yonginensis), Chryseolinea, Flexibacter, Arcicella, Dyadobacter, Emticicia (E. sediminis, E. paludis), Flectobacillus, Lacihabitans, Pseudarcicella, Runella* |
|  | 26 | Flavobacteriales | E |  |  |  |  | + |  |  | + | 456005 | *Fluviicola, Actibacter, Flavobacterium (F. terrigena, F. psychrolimnae, F. aquatile, F. cheniae, F. sinopsychrotolerans, F. collinsense, F. urocaniciphilum, F. paronense, F. swingsii, F. anseonense, F. dankookense, F. sasangense), Chryseobacterium* |
|  | 27 | Sphingobacteriales | W |  |  |  |  | + | + |  |  | 187623 | *Pedobacter (P. daechungensis, P. koreensis, P. insulae, P. aquaticus), Solitalea, Sphingobacterium* |
|  | 28 | Ignavibacteriales | E |  |  |  |  |  |  | + |  | 845 |  |
|  | 29 | Kapabacteriales | W |  |  |  | + |  | + |  |  | 4932 | *Kapabacteriales* |
|  | 30 | SJA-28 | E |  |  |  |  |  |  |  |  | 550 |  |
| Bdellovibrionota | 31 | Bacteriovoracales | W |  | + |  |  |  | + |  |  | 2563 | *Peredibacter* |
|  | 32 | Bdellovibrionales | W |  |  |  | + |  |  | + |  | 18176 |  |
|  | 33 | Silvanigrellales | E |  |  |  |  |  |  |  | + | 1146 |  |
| Campylobacterota | 34 | Campylobacterales | W |  |  |  |  | + |  | + |  | 21079 | *Arcobacter, Pseudarcobacter, Sulfuricurvum* |
| Chloroflexi | 35 | Chloroflexales | W |  |  |  | + |  | + |  |  | 7034 | *Herpetosiphon* |
|  | 36 | SL56_marine_group | W |  |  |  |  |  |  | + |  | 2954 |  |
| Cyanobacteria | 37 | unidentified in Class Cyanobacteriia | E |  |  |  |  |  |  | + |  | 667 |  |
|  | 38 | Chloroplast | W |  | + |  |  |  | + |  |  | 1248323 |  |
|  | 39 | Cyanobacteriales | E | + |  |  |  |  | + |  |  | 72588 | *Chamaesiphon PCC-6605, Microcoleus PCC-7113, Planktothrix NIVA-CYA 15, Tychonema CCAP 1459-11B, Chroococcidiopsis PCC-6712, Chroococcopsis (C. gigantea), Pleurocapsa PCC-7319, Pleurocapsa PCC-7319 (P. minor)* |
|  | 40 | Leptolyngbyales | E |  |  |  |  |  |  |  |  | 12366 | *Chamaesiphon PCC-7430 (C. investiens, C. subglobosus)* |
|  | 41 | Oxyphotobacteria_Incertae_Sedis | E |  |  |  |  |  |  |  |  | 6746 | *Calothrix KVSF5,*  *Phormidium CYN64* |
|  | 42 | Phormidesmiales | E |  |  |  |  |  |  |  | + | 736 | *Phormidesmis ANT.LACV5.1* |
|  | 43 | Pseudanabaenales | E |  |  |  |  |  |  | + |  | 8386 | *Pseudanabaena PCC-6802* |
|  | 44 | SepB-3 | E |  |  |  |  |  |  |  | + | 8024 |  |
|  | 45 | Synechococcales | E |  |  |  | + |  |  |  | + | 20016 | *Cyanobium PCC-6307, Schizothrix LEGE 07164* |
|  | 46 | Sericytochromatia | E |  | + |  |  |  |  | + |  | 837 | *Sericytochromatia* |
|  | 47 | Vampirovibrionales | E |  |  |  |  |  |  | + |  | 741 |  |
| Deinococcota | 48 | Deinococcales | E |  |  |  |  |  |  |  |  | 7983 | *Truepera* |
| Desulfobacterota | 49 | Desulfuromonadia |  |  |  |  |  |  |  |  |  | 785 | *Geothermobacter* |
|  | 50 | Geobacterales |  |  | + |  |  |  |  | + |  | 961 |  |
|  | 51 | PB19 | E |  |  |  | + |  |  |  |  | 492 |  |
|  | 52 | Syntrophales | E |  |  |  |  |  |  |  |  | 721 |  |
| Firmicutes | 53 | Bacillales | E |  |  |  |  |  |  |  | + | 2296 | *Bacillus, Paenisporosarcina* |
|  | 54 | Exiguobacterales | E |  |  |  |  |  |  |  | + | 8854 | *Exiguobacterium (E. undae)* |
|  | 55 | Lactobacillales | E |  |  |  |  | + |  |  | + | 1226 | *Trichococcus* |
|  | 56 | Clostridiales |  |  |  |  |  | + |  |  |  | 744 | *Clostridium (C. chromiireducens)* |
|  | 57 | Peptostreptococcales-Tissierellales |  |  |  |  |  | + |  |  |  | 2122 | *Fusibacter, Romboutsia* |
| Fusobacteriota | 58 | Fusobacteriales | W |  |  |  |  | + |  | + |  | 534 | *Hypnocyclicus* |
| Gemmatimonadota | 59 | Gemmatimonadales | E |  | + |  |  |  | + |  |  | 4212 | *Gemmatimonas* |
| Myxococcota | 60 | Myxococcales | W |  |  |  | + |  |  | + |  | 592 |  |
|  | 61 | Blfdi19 | E |  | + |  |  |  | + |  |  | 517 |  |
|  | 62 | Haliangiales | E |  |  |  |  |  |  | + |  | 763 | *Haliangium* |
|  | 63 | Polyangiales | E |  |  |  |  |  | + |  |  | 1735 | *Aetherobacter (A. rufus)* |
| NB1-j | 64 | NB1-j | E |  |  |  |  |  |  | + |  | 1047 |  |
| Nitrospirota | 65 | Nitrospirales | E |  |  |  |  |  |  | + |  | 26581 | *Nitrospira (N. moscoviensis)* |
|  | 66 | uncultured in Class Thermodesulfovibrionia | E |  |  |  |  |  |  |  |  | 896 |  |
| Patescibacteria | 67 | Absconditabacteriales_(SR1) | E |  |  |  |  | + |  |  | + | 812 |  |
|  | 68 | Gracilibacteria | W |  |  |  |  | + | + |  |  | 2508 | *Gracilibacteria* |
|  | 69 | unidentified in Class Parcubacteria | E |  |  |  |  | + |  | + |  | 4055 |  |
|  | 70 | Parcubacteria | E |  |  |  |  |  |  |  | + | 590 | *Parcubacteria* |
|  | 71 | Saccharimonadales | E |  |  |  |  | + |  |  | + | 12325 |  |
| Proteobacteria | 72 | unidentified in Class Alphaproteobacteria | E |  |  |  |  |  |  |  | + | 2559 |  |
|  | 73 | Acetobacterales | W |  |  |  | + |  |  |  |  | 2585 | *Roseomonas* |
|  | 74 | Caulobacterales | E |  | + |  |  |  |  |  | + | 24283 | *Brevundimonas, Caulobacter (C. fusiformis), Hirschia, Hyphomonas* |
|  | 75 | Defluviicoccales | E |  |  |  |  |  |  |  |  | 485 |  |
|  | 76 | Dongiales | E |  |  |  |  |  |  |  | + | 950 | *Dongia* |
|  | 77 | Holosporales | W |  | + |  |  |  |  | + |  | 745 |  |
|  | 78 | Micropepsales | E |  |  |  |  |  | + |  |  | 810 |  |
|  | 79 | Paracaedibacterales | W |  | + |  |  |  |  |  |  | 698 |  |
|  | 80 | Rhizobiales | E |  |  |  | + |  |  |  | + | 55319 | *Bosea, Devosia, Hyphomicrobium (H. vulgare), Pedomicrobium, Phreatobacter, Bradyrhizobium* |
|  | 81 | Rhodobacterales | E |  |  |  |  |  |  |  | + | 64708 | *Flavimaricola, Pseudorhodobacter, Rhodobacter, Tabrizicola* |
|  | 82 | Rhodospirillales |  |  |  |  |  |  |  | + |  | 625 |  |
|  | 83 | Rickettsiales | E |  |  | + |  |  |  | + |  | 6631 | *Rickettsia (R. limoniae)* |
|  | 84 | SAR11_clade | W |  |  |  | + |  |  |  |  | 85753 |  |
|  | 85 | Sphingomonadales | E |  |  |  |  | + |  |  | + | 360423 | *Altererythrobacter (A. palmitatis, A. buctensis), Novosphingobium (N. fuchskuhlense), Parablastomonas, Polymorphobacter, Porphyrobacter, Rhizorhapis, Sandarakinorhabdus (S. limnophila), Sphingomonas (S. fonticola), Sphingopyxis, Sphingorhabdus (S. wooponensis, S. planktonica, S. rigui), Sphingosinicella* |
|  | 86 | unidentified in Class Gammaproteobacteria | E |  |  |  |  |  |  | + |  | 2293 |  |
|  | 87 | 211ds20 | W |  |  | + | + |  |  | + |  | 2352 |  |
|  | 88 | AT-s2-59 | E |  |  |  |  |  |  |  |  | 3618 |  |
|  | 89 | Aeromonadales | E |  |  |  |  |  |  | + |  | 183777 | *Aeromonas (A. bivalvium), Tolumonas* |
|  | 90 | Alteromonadales | E |  |  |  | + |  |  | + |  | 68515 | *Rheinheimera (Pararheinheimera tilapiae, Pararheinheimera soli,*  *Pararheinheimera chironomi,*  *Pararheinheimera texasensis,*  *Pararheinheimera tangshanensis), Shewanella* |
|  | 91 | Burkholderiales | W |  |  |  |  | + |  |  |  | 2099003 | *Achromobacter (A. marplatensis), Rivicola (R. pingtungensis), Lautropia, Limnobacter (L. thiooxidans), Polynucleobacter (P. acidiphobus, P. cosmopolitanus, P. duraquae, P. difficilis), Chitinibacter (C. suncheonensis), Deefgea, Iodobacter, Chitinimonas (C. taiwanensis), Chitinivorax, Vogesella (V. indigofera, V. lacus), Acidovorax, Aquabacterium, Comamonas (C. jiangduensis), Delftia, Curvibacter, Hydrogenophaga, Ideonella, Inhella, Leptothrix (L. cholodnii), Limnohabitans (L. parvus, L. curvus, L. australis), Malikia (M. spinosa), Methylibium, Paucibacter, Polaromonas (P. naphthalenivorans, P. eurypsychrophila), Rhizobacter, Rhodoferax, Sphaerotilus, Sphaerotilus, Tibeticola, Thiobacillus, Methylotenera, Nitrosomonas, Duganella, Massilia, Pseudoduganella, Rugamonas (R. rubra), Undibacterium (U. parvum, U. pigrum), Azospira, Dechloromonas (D. hortensis), Ferribacterium, Sulfurisoma, Sulfuritalea, Thauera, Zoogloea (Z. ramigera, Z. oryzae), Sulfuricella* |
|  | 92 | CCD24 | E |  |  |  |  |  |  | + |  | 826 |  |
|  | 93 | CCM19a | E |  |  |  |  |  |  |  |  | 2117 |  |
|  | 94 | Cellvibrionales | E |  |  | + |  |  |  |  |  | 30672 | *Cellvibrio (C. gandavensis)* |
|  | 95 | Diplorickettsiales | W |  |  |  |  | + |  |  |  | 1170 | *Rickettsiella* |
|  | 96 | Enterobacterales | E |  |  |  |  | + |  | + |  | 10854 |  |
|  | 97 | Ga0077536 | E |  |  | + |  |  |  | + |  | 749 |  |
|  | 98 | Gammaproteobacteria_Incertae_Sedis | E |  |  |  | + |  |  | + |  | 14872 | *Acidibacter* |
|  | 99 | KF-JG30-C25 |  |  |  |  |  |  |  | + |  | 534 |  |
|  | 100 | KI89A_clade | E |  |  |  |  |  |  | + |  | 2173 |  |
|  | 101 | Legionellales | W |  |  |  | + |  |  |  |  | 605 | *Legionella* |
|  | 102 | Methylococcales | E |  |  |  |  | + |  | + |  | 11326 | *Crenothrix, Methylobacter (M. tundripaludum), Methyloglobulus (M. morosus)* |
|  | 103 | Oceanospirillales | W |  |  | + |  |  | + |  |  | 14594 | *Halomonas, Pseudohongiella* |
|  | 104 | PLTA13 | E |  |  |  |  |  |  |  |  | 2195 |  |
|  | 105 | Pseudomonadales | E |  |  |  | + |  |  |  |  | 622156 | *Acinetobacter (A. kyonggiensis, A. harbinensis, A. albensis, A. celticus, A. towneri), Alkanindiges, Cavicella, Fluviicoccus (F. keumensis), Pseudomonas (P. linyingensis, P. pohangensis)* |
|  | 106 | R7C24 | E |  | + |  |  |  |  | + |  | 938 |  |
|  | 107 | Steroidobacterales | E |  | + |  |  |  | + |  |  | 16587 | *(Hypsibius dujardini), Woeseia* |
|  | 108 | Thiotrichales | W |  |  |  |  | + |  |  |  | 595 | *Thiothrix (T. eikelboomii)* |
|  | 109 | Xanthomonadales | E |  |  | + |  |  |  |  | + | 205328 | *Ahniella (A. affigens), Dokdonella, Tahibacter, Arenimonas (A. subflava, A. metalli, A. daechungensis), Lysobacter (L. oligotrophicus, L. hankyongensis), Silanimonas, Stenotrophomonas, Thermomonas (T. carbonis)* |
| Verrucomicrobiota | 110 | Chthoniobacterales | W |  | + |  |  |  | + |  |  | 22804 | *Chthoniobacter, Terrimicrobium* |
|  | 111 | Methylacidiphilales | W |  |  |  |  |  |  | + |  | 864 |  |
|  | 112 | Opitutales | W |  |  | + |  |  |  | + |  | 39155 | *Cephaloticoccus, Lacunisphaera, Opitutus* |
|  | 113 | Pedosphaerales | W |  |  |  | + |  |  |  |  | 17801 |  |
|  | 114 | Verrucomicrobiales | E |  | + |  |  |  | + |  |  | 110471 | *Luteolibacter (L. arcticus, L. algae), Rubritalea, Brevifollis, Prosthecobacter, Verrucomicrobium* |
|  | 115 | unidentified in Class Verrucomicrobiae uncultured | W |  |  |  |  |  |  | + |  | 209060 |  |
